# Myocardial area at risk and salvage measured by T2-weighted cardiovascular magnetic resonance: Reproducibility and comparison of two T2-weighted protocols

**DOI:** 10.1186/1532-429X-13-50

**Published:** 2011-09-15

**Authors:** Jacob Lønborg, Niels Vejlstrup, Anders B Mathiasen, Carsten Thomsen, Jan S Jensen, Thomas Engstrøm

**Affiliations:** 1 Department of Cardiology, Rigshospitalet, Copenhagen, Denmark; 2 Department of Radiology, Rigshospitalet, Copenhagen, Denmark; 3 Department of Cardiology, Gentofte Hospital, Copenhagen, Denmark

**Keywords:** Cardiovascular magnetic resonance, area at risk, T2-weighted imaging, ST-segment elevation myocardial infarction, reproducibility

## Abstract

**Background:**

Late Gadolinium Enhancement (LGE) and T2-weighted cardiovascular magnetic resonance (CMR) provides a means to measure myocardial area at risk (AAR) and salvage. Several T2-weighted CMR sequences are in use, but there is no consensus in terms of which sequence to be the preferred. Therefore, the aim of the present study was to: (1) Assess the reproducibility and (2) compare the two most frequently used T2-weighted CMR protocols for measuring AAR and salvage.

**Methods:**

91 patients with ST-elevation myocardial infarction treated with primary percutaneous coronary intervention underwent a CMR scan 1-7 days after initial treatment. Two different T2-weighted protocols, varying in slice thickness and echo time (TE), were applied covering the entire left ventricle (LV) (protocol 1: TE 65 msec and slice thickness 15 mm; protocol 2: TE 100 msec and slice thickness of 8 mm). On a second scan performed 3 months later, infarct size was assessed with a standard LGE sequence. The two protocols were compared in terms of AAR and salvage index. Furthermore, intra- and interobserver reproducibility were assessed.

**Results:**

Protocol 1 measures a larger AAR and salvage index than protocol 2 with a mean difference in AAR of 1 ± 8%LV (p < 0.01) and 6 ± 12 g (p < 0.01) and salvage index of 0.04 ± 0.12 (p < 0.01). Both protocols had a high intra- and interobserver reproducibility with acceptable limits of agreement (6-8%LV and 6-12 g in AAR and 0.06-0.08 in salvage index).

**Conclusions:**

We report acceptable reproducibility for AAR and salvage index measured by T2-weighted images. Thus CMR is a reliable tool for measuring AAR and salvage index. Protocol 2 (8 mm slice thickness and 100 msec TE) measures slightly smaller AAR than protocol 1 (15 mm slice thickness and 65 msec TE), but the present study does not allow for a clear recommendation of either of the protocols.

## Background

In patients with acute and chronic myocardial infarction Late Gadolinium Enhancement (LGE) cardiovascular magnetic resonance (CMR) provides an accurate method for measuring myocardial infarct size[1]. This method has proven superior to other modalities such as single photon emission computed tomography (SPECT) in terms of detection of infarction and reproducibility[2-4]. CMR has therefore been used increasingly to measure infarct size as endpoint in clinical trials. However, in order to evaluate the effect of a given reperfusion therapy in patients with acute myocardial infarction the assessment of myocardial area at risk (AAR) and myocardial salvage provides important additional information. The AAR is defined as the part of the myocardium that is endangered during an acute occlusion of a coronary artery.

Even short periods of ischemia lead to accumulation of fluid and edema in the ischemic myocardium[5]. Using a T2-weighted short tau inversion recovery (T2-STIR) turbo spin echo sequence it is possible to visualize edema in the myocardium[6-8], which has been found comparable to histopathological[9] and fluorescein measurement of AAR[6]. Recently, Carlsson et al. found that the AAR size measured on T2-weighted images was comparable to the size of AAR measured using SPECT[10]. Thus, CMR has the potential to determine the myocardial infarct size, AAR and salvage myocardium. Today, different T2-weighted images protocols are in use with varying times to echo (TE) and slice thickness. The two most frequently used protocols have been TE 65 ms and slice thickness 15 mm[11-14], and TE 100 ms and slice thickness 6-10 mm[15-21]. Quantitative assessment of the AAR and salvage using T2-weighted images reproducibility has not been established and currently there is no standardized T2-weighted CMR protocol for assessing AAR in patients with acute myocardial infarction.

The aim of the present study was to: (1) Assess the reproducibility of T2-weighted CMR images and (2) compare the two most frequently used T2-weighted protocols in terms of size of AAR and myocardial salvage in patients with ST-segment elevation myocardial infarction (STEMI).

## Method

### Study population

Patients were enrolled if they had a STEMI of less than 12 hours duration from onset of symptoms until arrival at the Department of Cardiology, Rigshospitalet, and were ≥ 18 years old. STEMI was defined as ST-segment elevation in 2 contiguous electrocardiographic (ECG) leads of > 0.1 mV in V_4 _- V_6 _or leads II, III and aVF, or > 0.2 mV in lead V_1 _- V_3_. The patients were not considered for enrolment if they presented with cardiogenic shock, previous myocardial infarction, stent thrombosis, unconsciousness, renal insufficiency (creatinine > 200 μm/l) or previous coronary artery bypass graft surgery. Furthermore, patients in whom biomarkers did not confirm the diagnosis of acute myocardial infarction were excluded from the study. Patients, eligible for primary percutaneous coronary intervention (pPCI), were pre-treated with aspirin (300 mg orally or 500 mg intravenously), clopidogrel (600 mg orally) and heparin (10.000 units intravenously). At admission coronary angiography was performed to identify the culprit lesion. Reflow was established by introducing a guidewire, thrombectomy and/or inflating a small size balloon (1.5-2.5 mm). The choice of stent was left to the operator, as was the decision of direct stenting. Balloon angioplasty alone was only allowed if a stent could not be deployed or was considered harmful. Treatment with glycoprotein IIb/IIIa receptor antagonists was administered if no contraindications were present. All patients were treated with aspirin 75 mg daily lifelong and clopidogrel 75 mg daily for 12 months.

### CMR

The patients were screened for contraindications for CMR, and if none existed a CMR scan was performed during index admission (from day 1 to day 7 after pPCI). A second scan was performed 3 months after discharge (90 ± 21 days after pPCI). The scans were performed on a 1.5 T scanner (Avanto scanner, Siemens, Erlangen, Germany) using a 6-channel body array coil. In order to measure edema and AAR two different T2-weighted sequences were applied on the first scan, in which the TE and slice thickness were 65 msec and 15 mm (standard STIR sequence on Siemens systems); and 100 msec and 8 mm (standard STIR sequence on Philips systems). The other imaging parameters were identical in the two protocols: Slice gaps were 0 mm, resolution matrix 192 × 192, field of view 300 - 360 mm, inversion time 180 msec, repetition time 2 R to R intervals. TE and inversion time were fixed for both protocols and not adjusted during the scan. A surface coil intensity correction was applied prior to both protocols. Multiple slices in the short-axis image plane were acquired to cover the entire left ventricle (LV). The first short axis slice was applied at the base of the heart, defined as the atrioventricular (AV) plane, in the diastolic time frame in the 4-chamber image view. Subsequent slices were stacked consecutively from the AV plane until the apex was imaged. Left ventricular ejection fraction (LVEF) was assessed by CMR on an ECG triggered balanced steady-state free procession cine sequence by applying multiple slices in the short-axis image plane covering the entire LV.

A second CMR scan was performed 3 months after the first scan to assess myocardial infarct size using LGE CMR[22-25]. The images were obtained 8-10 minutes after intravenous injection of 0.1 mmol/kg body weight gadolinium-diethlenetriamine pentaacetic acid (Gadovist, Bayer Schering, Berlin, Germany). A standard ECG triggered inversion-recovery sequence was used (slice thickness 8 mm, slice gap 0 mm, TE 1.4 msec, resolution matrix 192 × 192, field of view 300 - 360 mm). In a single slice the inversion time was adjusted to null signal from normal myocardium, after which time multiple slices in the short-axis image plane was acquired covering the entire LV.

### Image Analysis

T2-weighted images were analyzed using ARGUS post-processing tool (ARGUS, Siemens, Erlangen, Germany). The endocardial and epicardial borders were manually traced in each short axis image and the LV myocardial volume was calculated. Papillary muscles and slow flowing blood in the trabeculae were carefully excluded from the LV myocardial volume. In order to assess the AAR, defined as a hyperintense area on T2-weighted images, the signal intensity (SI) in the normal (remote) myocardium was determined in each slice by tracing a region of interest (ROI) of at least 10 pixels within the visually normal myocardium. However, to avoid including areas of low SI (artefacts), several areas (three) were traced in the visually normal myocardium and the area with the highest SI was chosen to define normal SI. These ROI's were traced with care not to include epicardium or endocardium. A myocardial area was regarded as hyperintensive, when the SI was more than 2 standard deviations above the SI in the normal myocardium. The image window was adjusted to this threshold (normal SI + 2 standard deviations) and the volume of the hyperintensive areas was manually traced and added up. Small areas of hyperintensity scattered throughout the normal myocardium were not considered a part of AAR. For each patient AAR was expressed as a percent of total LV myocardial volume (%) and as absolute mass (g) using a density of 1.05 g/ml. Hypointensive areas within the AAR (haemorrhage or microvascular obstruction) were considered a part of AAR[15,18]. Hyperintensity in the blood pool from slow flowing blood adjacent to the endocardium was carefully excluded. This algorithm was used for both protocols. Furthermore, the quality of the images was evaluated and if one or more images were insufficient for analysis, the whole examination was registered as insufficient quality for analysis. One observer (JL) analyzed the images blinded without reviewing the result from the other protocol.

In order to assess intra- and interobserver reproducibility, two observers (JL and ABM) blinded to previous results analyzed the images in 20 patients 7 months after than the initial analysis. These 20 patients were chosen arbitrarily as the ones included in the middle of the inclusion period. Blinding was achieved by presenting the images to the observers without reviewing the results from either the same observer (interobserver,) the other observer (intraobserver) or the other protocol.

LGE CMR images was analyzed using the freely available software Segment v1.8 http://segment.heiberg.se[26]. The infarct size, defined as the hyperenhanced myocardium on the LGE images, was determined by a semi-automatic weighted approach. Endocardial and epicardial borders were manually traced in each short axis image and LV myocardial mass was calculated. Papillary muscles were accounted as part of the cavity. Infarct size was express as a percent of the total LV myocardial mass. Myocardial salvage index was calculated as follows: (AAR (g) - infarct size (g))/AAR (g). On cine short axis CMR images the LV volume was calculated in all 25 phases by manually tracing the endocardial borders. The diastolic and the systolic frames were automatically identified according to the size of the LV blood pool area, and LVEF was calculated accordingly. Papillary muscles were included in LV lumen. The analysis was performed with an ARGUS post-processing tool (ARGUS, Siemens, Erlangen, Germany).

For each protocol in each CMR examination the T2-weighted image with the largest area of hyperintensity were selected for signal-to-noise ratio (SNR) and contrast-to-noise ratio (CNR) analyses. On the selected image the SI was measured in the normal myocardium, in the region with hyperintensity and outside the body anterior to the chest wall (noise). CNR was calculated for each protocol as follows: (SI_hyperintensity_-SI_normal_)/SI_noise_. SNR was calculated in normal and hyperintensive myocardium for each protocol as follows: SI_hyperintensity_/SI_noise _and SI_normal_/SI_noise._

### Statistical analysis

The total numbers of insufficient examinations were compared using chi^2 ^test. The two protocols were compared in terms of AAR and salvage index with a paired t-test and visually by Bland Altman plots. Normal distribution was assumed for the variables in the Bland Altman plots. Differences between the two protocols and intra- and interobserver reproducibility were expressed as mean difference (± limit of agreement). Limits of agreement was calculated as 2 * standard deviation of the difference. CNR and SNR were compared using paired t-test. Continuous variables are expressed as mean ± standard deviation unless otherwise indicated. A two-sided p-value < 0.05 was considered statistically significant. All analysis was performed with SPSS software version 17 (SPSS inc., Chicago, Illinois, USA).

## Results

In this study a total of 91 patients were included, of which 12 patients did not show up for the second scan and therefore were not evaluated in terms of salvage index. The patient characteristics are shown in Table 1.

**Table 1:** Baseline clinical and angiographic characteristics

	Patients (n = 91)
Age, y	60 ± 11
Male gender, (%)	72 (79)
BMI, kg/m^2^	27 ± 4
Diabetes, (%)	6 (7)
Hypertension, (%)	24 (26)
Hypercholesterolaemia, (%)	47 (52)
Previous PCI, (%)	3 (3)
Culprit lesion, (%)	
RCA	36 (40)
LAD	35 (39)
Cx	20 (21)
Time to pPCI, minutes	165 (120-275)
Time to CMR 1, days	1 (1-2)
Time to CMR 2, days	90 (85-93)
LVEF, %	53 ± 8

Data are expressed as mean ± standard deviation, median (IQR) or n (%) unless otherwise is indicated.

BMI, body mass index; CMR, cardiac magnetic resonance; Cx, left circumflex artery; LAD, left anterior descending artery; LVEF, left ventricular ejection fraction; pPCI, primary percutaneous coronary intervention; RCA, right coronary artery.

### Reproducibility

Intra- and interobserver reproducibility are summarised in Table 2 and was over-all high. The variability was lowest for AAR expressed as %LV for both protocols with mean bias ranging 0-1%LV and limits of agreement ranging ± 6-8%LV. Whereas, variability for both protocols were higher for AAR expressed as grams with mean bias ranging 0-6 g with limits of agreement ranging ± 6-12 g. Salvage index had mean bias of 0.00-0.03 with limits of agreement limits ± 0.06-0.08. Over-all the protocols have comparable reproducibility. The patients were further divided in two groups according to LVEF ≥ 50% (n = 12) or LVEF < 50% (n = 8). In the patients with normal or near normal LVEF (≥ 50%) intra- and interobserver limits of agreement for AAR ranged ± 4-6%LV and ± 6-10 g compared to ± 4-8%LV and ± 8-16 g in the patients with reduced LVEF (< 50%). These findings indicate a slightly better reproducibility in patients with normal or near normal LVEF.

**Table 2:** Intra- and interobserver reproducibility

	Protocol 1 (TE 65 msec; slice thickness 15 mm)	Protocol 2 (TE 100 msec; slice thickness 8 mm)
	
	Mean difference (± limits of agreement)	Mean difference (± limits of agreement)
Area at risk, %LV		
Interobserver reproducibility	0 (± 6)	0 (± 8)
Intraobserver reproducibility	0 (± 6)	1 (± 6)
Area at risk, g		
Interobserver reproducibility	-2 (± 10)	-6 (± 6)
Intraobserver reproducibility	0 (± 12)	-3 (± 10)
Salvage index		
Interobserver reproducibility	0.00 (± 0.08)	-0.03 (± 0.06)
Intraobserver reproducibility	0.01 (± 0.06)	-0.02 (± 0.08)

Mean difference (± limit of agreement = 2 × standard deviation)

LV, left ventricle; TE, time to echo.

### Comparison of the Protocols

**Figure 1:**
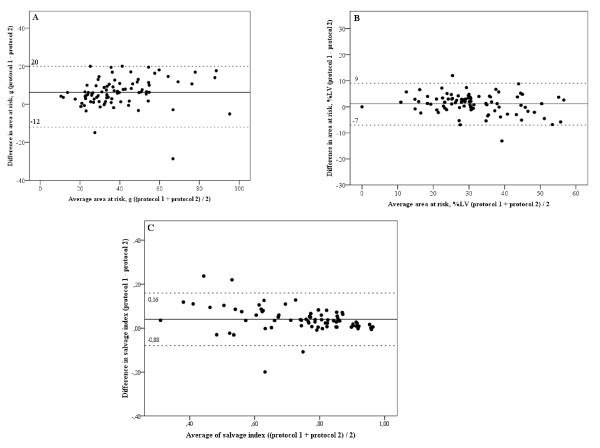
**Bland Altman plot of the mean difference between the two different protocols**. This figures shows the mean difference between protocol 1 (TE 65msec; slice thickness 15 mm) and protocol 2 (TE 100 msec; slice thickness 8 mm) in area at risk expressed as % of the left ventricle (LV) **(A) **and in absolute mass (g) **(B) **and in salvage index **(C)**. The marked line represents the mean difference and the dotted lines represent upper and lower limits of agreement. LV, left ventricle.

**Figure 2:**
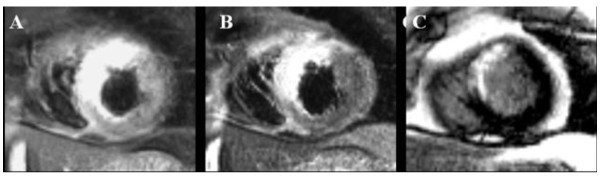
**T2-weighted and LGE CMR images**. T2-weighted cardiac magnetic resonance (CMR) images acquired by (A) protocol 1 (TE 65msec; slice thickness 15 mm) and (B) protocol 2 (TE 100 msec; slice thickness 8 mm) and (C) LGE CMR in a 62-years-old male with anteroseptal infarction due to occlusion of the left anterior coronary artery. The myocardial infarct size was 9%LV (left ventricle). The myocardial area at risk was 31%LV measured by protocol 1 and 28%LV by protocol 2.

**Figure 3:**
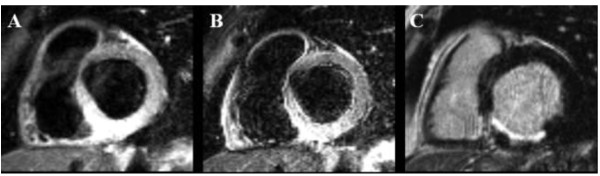
**T2-weighted and LGE CMR images**. T2-weighted cardiac magnetic resonance (CMR) images acquired by (A) protocol 1 (TE 65msec; slice thickness 15 mm) and (B) protocol 2 (TE 100 msec; slice thickness 8 mm) and (C) LGE CMR in a 64-years-old male with posterior infarction due to occlusion of the right coronary artery. The myocardial infarct size was 8%LV (left ventricle). Myocardial area at risk was and 31%LV measured by protocol 1 and 26%LV by protocol 2.

The image quality was insufficient for analysis using both protocols in three patients and in four additional patients using protocol 2, but the difference in the rate of insufficient scans between the two protocols did not reach statistical significance (3 in protocol 1 versus 7 in protocol 2, p = 0.17). AAR expressed as %LV (protocol 1: 32%LV ± 11 versus protocol 2: 31%LV ± 11; p < 0.01) and as grams (protocol 1: 43 g ± 18 versus protocol 2: 37 g ± 17; p < 0.01) were significant larger measured by protocol 1 than with protocol 2. In the bland-Altman plots more marks were above zero that below, indicating that protocol 1 more frequently measures a larger AAR that protocol 2 (Figure 1A and 1B). Importantly, this observation seems to be independent of the size of the AAR, since the marks in the Bland Altman plot (Figure 1A and 1B) are distributed equally along the x-axis. The Bland Altman analysis revealed a systematic bias between protocol 1 and 2 with a mean difference of 1%LV (limits of agreement ± 8 g) and 6 g (limits of agreement ± 14 g). Examples of images acquired by the two protocols are illustrated in Figure 2 and 3.

Protocol 1 measured a larger salvage index than protocol 2 (protocol 1: 0.76 ± 0.14 versus protocol 2: 0.72 ± 0.16; p < 0.01). The Bland Altman plot revealed a systematic bias between protocol 1 and 2 in terms of salvage index with a mean difference of 0.04 and limits of agreement ± 0.12 (Figure 1C).

### Contrast-to-noise and signal-to-noise

No statistical difference in CNR was observed between protocol 1 and protocol 2 (41 ± 43 versus 36 ± 43; p = 0.12). Whereas, protocol 1 had a significant higher SNR in the AAR (127 ± 108 versus 81 ± 85; p < 0.01) and a higher SNR in the normal myocardium (86 ± 68 versus 46 ± 46; p < 0.01).

## Discussion

To our best knowledge, this is the first study to compare the size of AAR and salvage index and reproducibility of the two mostly used T2-weighted protocols. The main findings were that both protocols from a clinical perspective had acceptable intra- and interobserver reproducibility and protocol 1 measures a slightly higher AAR and salvage index than protocol 2, but despite this systematically bias the protocols were overall comparable in terms of quantitative assessment of both parameters. Thus, T2-weighted images are a reliable method to measure AAR and salvage index.

### Reproducibility

In this study intra- and interobserver reproducibility were acceptable for both protocols. Reproducibility was higher for AAR expressed as a relative measure (%LV) compared to AAR expressed as an absolute measure (g) and protocol 2 has smaller limits of agreement for AAR expressed as absolute size (g) than protocol 1. These findings are most likely due to AAR as a relative measure is less dependent on precise and accurate endocardial and epicardial border detection and protocol 1 due to thicker slices leads to more signal from slow flowing blood in the trabeculae. Importantly, reproducibility for salvage index measured using CMR was high, which makes it an excellent surrogate end point in clinical trials. The currently most used method to detect AAR and salvage index is SPECT, but this method is limited by the uptake of the tracer, a tracer is injected intravenously before intervention and the scan can be performed up to 8 hours thereafter. T2-weighted images can on the other hand evaluate AAR up to 1 week after the ischemic event[10]. The reproducibility of AAR measured using SPECT is ± 6%LV,[27,28] which is similar to the reproducibility in the present study using CMR (± 6-8%LV). Furthermore, to the best of the authors' knowledge there exits no published data for AAR expressed as an absolute measure (g) using T2-weighted images or SPECT. Measuring AAR in grams using T2-weighted images seems to be less reproducible compared to calculating AAR as %LV. However, AAR is mostly used in relation to infarct size and to calculated myocardial salvage in order to evaluate the effect of a given intervention. Therefore, using AAR and salvage index as a measurement to evaluate a proof-of-concept it seems more relevant to focus on the myocardial region supplied by the occluded coronary vessel. Expressing AAR in grams compared to in %LV can thus be more interesting from a scientific perspective, but a lower reproducibility needs to be taken into account. Furthermore, findings in the present study indicate that the reproducibility of T2-weighted images is slightly higher in patients with normal or near normal LV function. This can be explained by the risk of motion artefacts related to the underlying segmental LV function, which probably is more pronounced in patients with LV dysfunction and reduced LVEF after STEMI. However, this finding must be taken with precaution due to the small sample size in each LVEF group and an unequal distribution of patients between the groups (n = 8 and n = 12).

Despite a high intra-and interobserver reproducibility in this study for both protocols and acceptable limits of agreement, the reproducibility was not perfect and not as high as the one previously reported for measurement of infarct size using LGE CMR[1,29]. The discrepancy yields the need for developing an automatic or semi-automatic approach when measuring AAR with T2-weighted images.

### Comparison of the Protocols

Using TE of 65 msec and slice thickness of 15 mm measured a slightly larger AAR and salvage index than using TE 100 msec and slice thickness 8 mm. Given infarct size was measured using a single method the choice of T2-weighted protocol directly impacted the calculation of the salvage index through differences in measured AAR. The discrepancy between the two protocols can be explained by the following: 1) Slow flowing blood trapped in the trabeculae, which is more pronounced using thicker slices and especially in regions of infarction due to hypokinesia and stunning. Despite that care is taken with regard to this issue, it may still be difficult to precisely distinguish between edema in the myocardium and slow flowing blood. Thus, using thicker slices probably leads to overestimation of the LV mass especially in the AAR; 2) The same algorithm was used for measuring AAR, which might disadvantage protocol 2, as a myocardial area had to have a stronger signal, than in protocol 1, to reach the +2 standard deviation threshold from normal myocardium; 3) The inherent inaccuracy analysing T2-weighted imaging may be as great as the intrinsic difference between the two protocols. However, the difference in AAR between the protocols does not alter clinical decision-making and given the lack of a relevant gold standard method for comparison it does not provide guidance for recommendation of either T2-weighted protocols.

The longer TE in protocol 2 compared to protocol 1 (100 msec versus 65 msec), gives an increased contrast, but also images with decreased SNR, which was further intensified by the choice of a slice thickness of 8 mm compared to 15 mm. These opposite effects gave a decreased CNR in protocol 2, but the difference did not reach statistical significance. Increasing the slice thickness also leads to increased signal from slow flowing blood in the ventricular lumen, is less sensitive towards detection of small focal defects and the endo- and epicardial border detection might be less precise. The latter issue is illustrated in the present study, since the limit of agreement was slightly larger for protocol 1 compared to protocol 2 in terms of AAR expressed as an absolute measurement. Using thinner slices might thus be more accurate due to less partial volume and less sensitive towards slow following blood in the trabeculae. Furthermore, CNR was not statistical significantly different between the two protocols in the present study. Taken together, due to lack of a gold standard method for comparison, findings in the present study do not allow for a clear recommendation for either of the T2-weighted protocols. However, the higher rate of insufficient scan obtained with protocol 2, though not statistically significant, may favour protocol 1. On the other hand, the slightly smaller limits of agreement of protocol 2 may favour this protocol. Finally, using thinner slices (protocol 2) may be preferable in patients with suspected dispersed focal pathology such as myocarditis and sarcoidosis.

As mentioned the detection of edema on T2-weighted images depends greatly on SI, SNR and CNR and is affected by cardiac motion. Alternative methods to detect edema that may overcome some of the problems regarding SI based assessment and through plane motion signal loss are emerging e.g. single short imaging with T2 prepared steady state free precession and T2 mapping[30,31]. However, results from previous studies using these methods are preliminary.

Previous studies that have used TE 65 msec and slice thickness 15 mm did only apply 3 slices to cover the LV (apical, mid and basal)[12-14,32], which might lead to inaccurate evaluation of the AAR. In the present study the whole LV was covered with no gaps, which may be a more accurate method for assessing the AAR.

### Study limitations

Scans were all performed on the same system and at a single centre, therefore results performed on another system and centre might be different. Another limitation is that the T2-weighted images from protocol 1 and 2 were analyzed consecutively, but also blinded to the final results from the other scan. Furthermore, reproducibility was assessed on images obtained from the same CMR examination. Thus, data in the present study does not take potential variances between two difference CMR examinations into account e.g. slice position, slice angling, heart rate and TI. In addition, this study only compares the T2-weighted protocols that to the authors best knowledge are the two most frequently used, but other protocols are in use; i.e. TE = 80 msec and slice thickness = 8 mm[33,34], and TE = 65 and slice thickness 10 mm[35]. Thus, the two used T2-weighted protocols in the present study are not truly representative of the field. Finally, the present study was performed under the assumption that edema on T2-weighted imaging represents AAR, which is based on previous validation studies[6,9,10]. However, these studies have their own inaccuracies, which warrants the need for comparison of T2-weighted imaging and edema with the gold standard method Evans Blue. Nevertheless, the purpose of the present study was not to validate T2-weighted imaging, but merely to look for consensus of the sequences and determine reproducibility.

## Conclusions

We report acceptable reproducibility for both T2-weighted protocols in terms of measuring AAR and salvage index. Thus CMR is a reliable tool for measuring AAR and salvage index. Protocol 2 (8 mm slice thickness and 100 msec TE) measures slightly smaller AAR than protocol 1 (15 mm slice thickness and 65 msec TE), but the present study does not allow for a clear recommendation of either of the protocols.

## Competing interests

The authors declare that they have no competing interests.

## Authors' contributions

All authors contributed to report writing, trial design, and the review of published work. JL contributed to writing of the study protocol, conceptual design, data quality control, and general data analysis; contributed to recruitment and enrollment of patients; carried out the magnetic resonance examinations and imaging analysis; and drafted the report and figures. NV contributed to conceptual design and data analysis; and magnetic resonance examinations and imaging analysis. ABM contributed to magnetic resonance examinations and imaging analysis. CT contributed to conceptual design; and magnetic resonance imaging analysis. JSJ contributed to conceptual design. TE was principal investigator; contributed to conceptual design; and contributed to recruitment and enrollment of patients, data quality control, and angiographic and general data analysis.
